# Proportional impact prediction model of coating material on nitrate leaching of slow-release Urea Super Granules (USG) using machine learning and RSM technique

**DOI:** 10.1038/s41598-024-53410-8

**Published:** 2024-02-06

**Authors:** Sidhartha Sekhar Swain, Tapan Kumar Khura, Pramod Kumar Sahoo, Kapil Atmaram Chobhe, Nadhir Al-Ansari, Hari Lal Kushwaha, Nand Lal Kushwaha, Kanhu Charan Panda, Satish Devram Lande, Chandu Singh

**Affiliations:** 1 Division of Agricultural Engineering, ICAR-Indian Agricultural Research Institute, New Delhi, 110012 India; 2 Division of Soil Science and Agricultural Chemistry, ICAR-Indian Agricultural Research Institute, New Delhi, 110012 India; 3 Department of Civil, Environmental and Natural Resources Engineering, Lulea University of Technology, 97187 Lulea, Sweden; 4 Department of Soil Conservation, National PG College (Barhalganj), DDU Gorakhpur University, Gorakhpur, UP 273402 India; 5 Division of Genetics, ICAR-Indian Agricultural Research Institute, New Delhi, 110012 India

**Keywords:** Environmental impact, Ecology, Environmental sciences

## Abstract

An accurate assessment of nitrate leaching is important for efficient fertiliser utilisation and groundwater pollution reduction. However, past studies could not efficiently model nitrate leaching due to utilisation of conventional algorithms. To address the issue, the current research employed advanced machine learning algorithms, viz., Support Vector Machine, Artificial Neural Network, Random Forest, M5 Tree (M5P), Reduced Error Pruning Tree (REPTree) and Response Surface Methodology (RSM) to predict and optimize nitrate leaching. In this study, Urea Super Granules (USG) with three different coatings were used for the experiment in the soil columns, containing 1 kg soil with fertiliser placed in between. Statistical parameters, namely correlation coefficient, Mean Absolute Error, Willmott index, Root Mean Square Error and Nash–Sutcliffe efficiency were used to evaluate the performance of the ML techniques. In addition, a comparison was made in the test set among the machine learning models in which, RSM outperformed the rest of the models irrespective of coating type. Neem oil/ Acacia oil(ml): clay/sulfer (g): age (days) for minimum nitrate leaching was found to be 2.61: 1.67: 2.4 for coating of USG with bentonite clay and neem oil without heating, 2.18: 2: 1 for bentonite clay and neem oil with heating and 1.69: 1.64: 2.18 for coating USG with sulfer and acacia oil. The research would provide guidelines to researchers and policymakers to select the appropriate tool for precise prediction of nitrate leaching, which would optimise the yield and the benefit–cost ratio.

## Introduction

Water is a vital and scarce resource in dryland agriculture. Farmers heavily rely on individual wells to pump groundwater for irrigation due to the dearth of surface water supplies and lack of proper irrigation. Groundwater quality is a topic that is receiving special attention due to its exposure to nitrate leaching^1^. One of the major non-point nitrate pollution causes is thought to be the loss of nutrients from agricultural areas through leaching and surface runoff of excess fertiliser^2^. Overfertilization, particularly of nitrogen (N) in highly productive agricultural areas, causes soil pollution, groundwater pollution and eutrophication of rivers and lakes^3,4^. This N surplus can stay in the soil or spread to other parts of the environment, causing a number of detrimental impacts^5^, such as soil acidification^6^ and air and water pollution^7^. The residual nitrate is very dynamic and mobile due to the negative charge of nitrate, which is similar to soil clay and can contaminate groundwater or surface water^8^.

A major challenge with nitrogen fertiliser is the poor nitrogen utilisation efficiency (NUE), which varies from 20 to 40% in lowland rice ecosystems^9,10^ and this loss results in excess urea application. Nowadays, a slow release of urea by the deep placement of urea super granule (USG) is used as an effective N management strategy. In China and Bangladesh, USG has been widely employed as the nitrogen release rate from USG is nearly equal to the nutrient intake rate of the crops^11,12^ Slow-release fertilisers such as USG have a very high nitrogen utilisation efficiency as the nutrient loss due to leaching, and volatilisation is minimised, and the metabolic requirements of plants are fulfilled.

The nutrient release could be further delayed by providing coating (sulfur, polymers, superabsorbent, and composites) to the USG. Utilising neem cake, neem-oil emulsion coated urea and Pusa neem golden urea, various experiments at the Indian Agricultural Research Institute, New Delhi, have shown an improvement in yields and apparent nitrogen recovery in rice^13,14^. Hoeung^15^ used bentonite clay as a binding agent at three different levels of 5%, 7.5%, and 10% and found that by increasing the levels of the binding agent, the release of nutrients was retarded. The nitrate release rate of USG when coated with neem oil, sulfur, and bentonite clay as a binding agent is yet to be studied. When the coated USG comes in contact with soil solution, the water (mostly vapour) initially permeates through the coating, which builds up internal pressure results^16,17^ and partially dissolves it^18–20^. After the coating is dissolved, the fertiliser gets released. The fertiliser discharge following the coating dissolution is a complex and nonlinear diffusion and mass transfer process which a linear model cannot explain.

Mathematical and analytical models have been used to solve those complex nonlinear diffusion and mass transfer processes^21–25^. wagenet^26^ used mathematical methods to describe the transport and transformation of urea into ${NH}_{4}^{+}$ and ${N0}_{3}^{-}$ in soil. The model relied on the assumption that pore water velocity, the apparent diffusion coefficient, and three rate constants (associated with urea hydrolysis, ammonium oxidation, and nitrate reduction) remained constant with depth and time. Notably, apparent diffusion coefficient and average pore water velocity exhibit considerable field variability, and the rate constants are subjected to diverse environmental influences, such as temperature, water content, organic carbon, and soil texture. The model acknowledges the complexity introduced by these factors but assumes constancy. However, uncertainties persist, especially regarding the cation exchange process, emphasizing the need for further research to refine our understanding of the intricate interactions between environmental variables and biogeochemical kinetics. These mechanistic models have limitations like over parameterisation, over sensitivity to changes in operating conditions and demand a lot of effort for calibration and validation^27^. The mechanistic model also has limitations in incorporating data from multiple data and time scales^28^. These limitations can be overcome by using machine learning (ML) algorithms. Conversely, ML is a data analysis tool that can learn from input data and make judgments without process equations or paths^29^. For prediction and classification tasks, ML algorithms identify a specific pattern based on prescribed data (input data) throughout a training process, producing a more accurate output^30,31^.

Artificial neural network (ANN) is one of the well-known soft computing techniques that has been created and used as a tool for problem-solving in various sectors. ANN has been used extensively in handling nonlinearity problems like groundwater level forecasting^32^, estimation of suspended load in flood conditions^33^, prediction of sediment load^34^, prediction for water quality index^35^, irrigation groundwater quality prediction^36^, and many others^37–48^. Another nonlinear regression method that handles outliers and nullifies overfitting issues is the support vector machine (SVM)^49,50^. SVM modelling demonstrates superior performance than standard machine learning techniques in most situations. It offers a lot of intriguing characteristics, such as an efficient avoidance of overfitting, which enhances its capacity to construct models utilising a high number of input variables and only a small number of experimental outcomes in the training set^51^. It has its uses in many fields, including the calculation of suspended sediment load^52,53^, water quality forecasting^54,55^, prediction of rainfall-runoff relationship^56–59^. Due to its capacity to prevent over-fitting, robustness to incorporate many input variable types without variable deletion and regularisation, and its exceptional analytical and operational flexibility, random forest (RF) has been characterised as an effective and robust method to solve nonlinear and complex processes^60^. It is a tree-based method consisting of non-parametric statistical approaches for conducting regression and classification analyses by recursive partitioning^61^. RF was used in the prediction of nonlinear processes like estimating soil cohesion^62^, prediction of soil organic matter content^63^, determination of water quality index^64^, prediction of groundwater pollution due to nitrate leaching^65^. Another method to solve the nonlinearity problem is the M5Tree model. The benefit of M5Tree over neural networks is that it offers straightforward rules and can be tuned more quickly. Additionally, its rules are simple to convey and apply in real-world situations^66^. M5Tree is an alternative data-driven strategy that is extremely clear and does not necessitate the optimisation of network geometry and internal parameters^67^. M5 model has been used in the prediction of nonlinear hydrological parameters like piezometric head and seepage studies^68^, computation of missing rainfall data^69^, sediment transportation^70^, modelling of crop evapotranspiration^71^, establishing water level-discharge relationship^72^, rainfall-runoff modelling^73^. Another ML model used in the hydrological field is the reduced error pruning tree (REPTree), a decision tree model developed by Breiman^74^. REPTree is used in solving various nonlinear and complex problems like the prediction of the spatial distribution of temperature of water across the dam^75^, prediction of meteorological drought^76^, modelling of landslide susceptibility^77^, modelling flood susceptibility^78^ etc.

Optimization of coating material is an important step which shows the composition of different coating material proportion for minimum nitrate leaching. Reduction in nitrate leaching has a lot of advantages in terms of water quality improvement^79,80^, enhanced public health^79,81^, reduction in climate change^82,83^, enhance in soil health and productivity^84,85^ and sustainability^81,86^ among others Now-a-days response surface methodology (RSM) is being used as an optimization technique in place of orthogonal statistics due to its efficiency, effectiveness, accuracy, robustness, and versatility. The second-order Box-Wilson central composite design (CCD) is the most well-liked and efficient RSM design. The CCD is ideal for integrating specific operation factors into a range of assessments since it uses a rationalised number of design points and a reliable curvature estimation to gather enough data to verify lack of fit^87^^,^^88^ optimized the teropolymer coating of urea for slow release of sulfer from coating using CCD design in RSM and found that 82.37% conversion of sulfer was achieved if 51.94% S is allowed to react with jatropha oil for 74.21 min. However there has been limited study on optimization of input parameters for coating of USG with an objective to reduce nitrate leaching.

In the light of the preceding reviews, the research gaps identified for the study wereAs per the best knowledge of the authors, to date, no study has been performed on the application of ML algorithms to release nitrate from USG, Nitrate release rate of USG when coated with neem oil, sulfur, and bentonite clay as a binding agent is yet to be studied and) optimization of coating parameters for optimum nitrate leaching has been less explored. Based on the above research gaps objectives of this study were determined as to develope machine learning models (ANN, SVM, RF, M5 Tree, REPTre and RSM) for prediction of nitrate release, followed by determination of nitrate release rate when the USG is coated with neem oil, sulfur, and bentonite clay and optimizing coating of USG for minimum nitrate leaching. The research work would help farmers, researchers and policymakers in proper coating of USG and precise prediction of nitrate leaching, which would enhance the profit of the farmers and reduction of environmental pollution.

## Materials and methods

### Coating of USG

Industrial grade USG (Nitrogen content 46%) having each granule weight 1.5 ± 0.2 gm was taken for this study. The coating was done in our laboratory. In this study, three types of coatings were done, i.e., USG with bentonite clay and neem oil without heat (T1) and USG with bentonite clay and neem oil with the application of heat (T2) and USG with sulphur and acacia oil (T3). For heat application, bentonite clay was heated to 80 °C, and then it was coated using neem oil. Required proportion of neem oil and nano bentonite clay were taken in a beaker and was coated manually by stirring using a glass rod. The manual coating process involved stirring the mixture with a glass rod until a visually uniform coating was achieved. For sulphur coating rotating drum was used^89^. Each of the three types of coating has 16 different compositions, as presented in Table 1. Then the coated products were kept to set for 1–5 days as curing period which has been termed as the age of the products.

**Table 1:** Composition of coating of urea.

Compositions	Bentonite clay and neem oil without heat (T1)			Bentonite clay and neem oil with heat (T2)			Sulphur and acacia oil (T3)		
	USG (gm)	Clay (gm)	Oil (ml)	USG (gm)	Clay (gm)	Oil (ml)	USG (gm)	Sulphur (gm)	Oil (ml)
C1	10	0.5	1	10	0.5	1	10	0.5	0.5
C2	10	0.5	2	10	0.5	2	10	0.5	1
C3	10	0.5	3	10	0.5	3	10	0.5	1.5
C4	10	0.5	4	10	0.5	4	10	0.5	2
C5	10	1	1	10	1	1	10	1	0.5
C6	10	1	2	10	1	2	10	1	1
C7	10	1	3	10	1	3	10	1	1.5
C8	10	1	4	10	1	4	10	1	2
C9	10	1.5	1	10	1.5	1	10	1.5	0.5
C10	10	1.5	2	10	1.5	2	10	1.5	1
C11	10	1.5	3	10	1.5	3	10	1.5	1.5
C12	10	1.5	4	10	1.5	4	10	1.5	2
C13	10	2	1	10	2	1	10	2	0.5
C14	10	2	2	10	2	2	10	2	1
C15	10	2	3	10	2	3	10	2	1.5
C16	10	2	4	10	2	4	10	2	2

### Laboratory analysis

**Figure 1:**
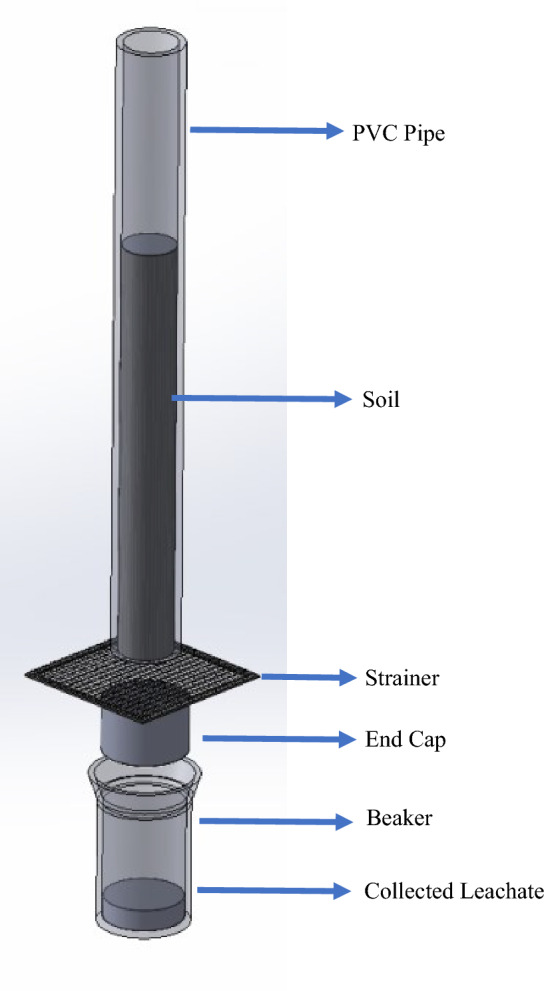
Pipe Setup for the laboratory study.

**Figure 2:**
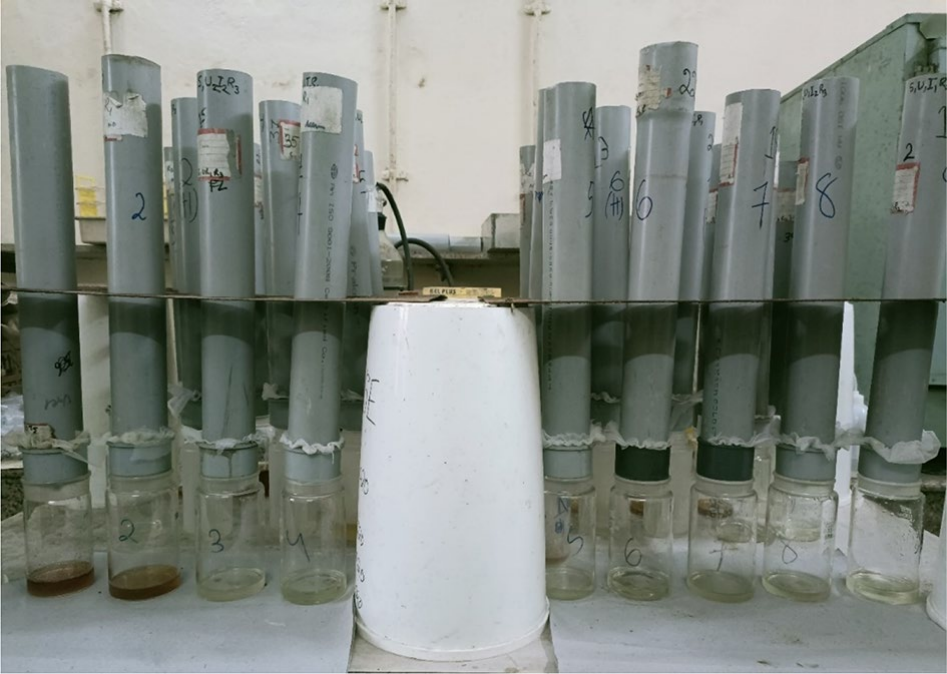
Laboratory set up for collection of leachate from the soil column.

For the leaching experiment purpose PVC pipes of 70 cm in length and 6.4 cm in internal diameter were used which can contain 1 kg soil. The soil used was sandy loam soil from paddy field for this study. These pipes are sealed at one end by an end cap with a hole in the centre. Net is placed between the pipe and end cap so only leachate, not soil, can pass through it. In each pipe 750 gm soil is put, then the treatments or control are placed, followed by 250 gm soil above it (Fig. 1). The soil was irrigated to saturation. The soil was irrigated up to the saturation moisture content and the leachate was collected in the beaker placed at the bottom of the column, as shown in Fig. 2.

**Figure 3:**
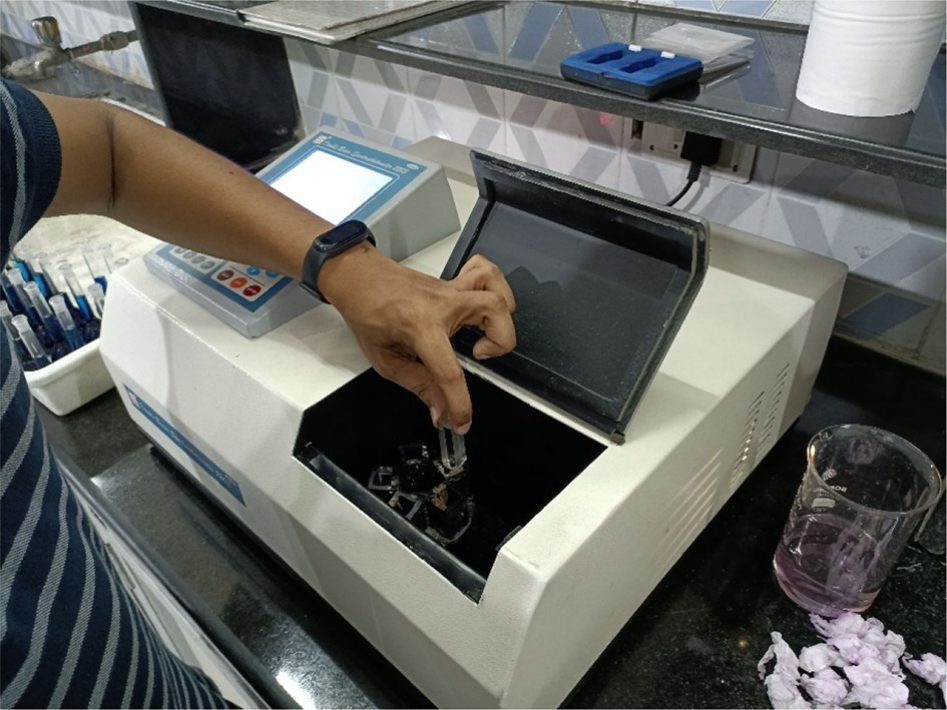
Determination of nitrate content by spectrophotometer.

Leachate was collected at an interval of 8 days for 32 days. The nitrate concentration of the leachate was calculated by the cadmium reduction method^90^. In this method, after the colour development of leachate, the absorbance of the aliquote was determined using a spectrophotometer at 540 nm against reagent blank solution, as shown in Fig. 3 Nitrate concentration was calculated by comparing the absorbance value with the standard value.

### Machine learning methods

Due to the ability to handle nonlinear correlations, high-order interactions, and non-normal data, machine learning technique has seen widespread usage in numerous ecological categorisation problems and predictive modelling^91–94^. The ml models used in this study are Artificial neural network (ANN), Support vector machine (SVM), M5P model tree (M5P), Random forest (RF), Reduced error pruning tree (REPTree) and response surface methodology (RSM), which are discussed further below.

#### Artificial neural network (ANN)

For computational research to forecast the response, ANNs were used. It has basic processing units called neurons, and each network contains artificial neurons arranged in layers and connected in parallel^95^. Artificial Neural Networks (ANNs) have three layers-input, hidden, and output-each with multiple neurons for non-linear computing. The hidden layer facilitates data transfer between input and output layers, conducting computations essential for functions like categorization and prediction. Like a feed-forward network, data in an ANN moves from the input to the output layer in the forward direction. ANNs excel in modeling nitrate leaching due to their capacity to capture complex non-linear relationships in environmental data^96^. Their adaptability suits dynamic systems, recognizing intricate patterns and interactions among variables^97^. Moreover, ANNs can handle missing data, ensuring meaningful predictions for nitrate leaching, making them effective tools for understanding and predicting the behavior of this environmental process.

#### Support vector machine (SVM)

To solve classification and regression problems, a supervised learning technique was developed by Vapnik^98^, known as a support vector machine (SVM). SVMs excel in modelling nitrate leaching due to their ability to capture complex non-linear patterns influenced by various environmental factors^99^. They perform well in high-dimensional spaces, handling the intricacies of systems with multiple variables. The kernel trick facilitates effective separation of classes in transformed spaces, maximizing the margin between them^100^. SVMs' robustness to outliers is advantageous in environmental datasets, and their generalization ability, with controlled overfitting, ensures reliable models for nitrate leaching, suitable for application to new data^101,102^.

#### M5P tree

A binary decision tree with a linear regression function at the terminal (leaf) nodes, such as the M5 model tree, may be used to predict continuous numerical properties. In order to develop tree-based models, a divide-and-conquer strategy is used. It is advantageous for modelling nitrate leaching due to its inherent interpretability^103^. By combining linear regression models in its leaves, M5P captures non-linear relationships crucial for understanding complex interactions among environmental factors. It provides insights into variable importance, aiding in identifying key factors influencing nitrate leaching. M5P's ability to handle datasets with multiple variables, ease of use, and potential for ensemble learning makes it accessible and effective for researchers and practitioners in environmental modelling^104^.

#### Random forest (RF)

In the RF approach, different decision tree algorithms are combined to generate repeated forecasts of the same phenomena. It can be used for both classification and regression. A major goal of this study is to forecast nitrate leaching so that regression mode will be the sole option offered in this section. It is well-suited for modelling nitrate leaching due to its ensemble learning approach, which handles non-linearity and complex interactions in environmental data^105^. It provides insights into variable importance, aiding in understanding significant factors. The algorithm is robust to overfitting, outliers, and high-dimensional data common in nitrate leaching modelling^106–108^.

#### Reduced error pruning tree (REPTree)

Fast learning is achieved using the REPTree algorithm. The decision/regression tree is constructed using information gain/variance and then pruned using reduced error with back-fitting. Reptree, a decision tree algorithm, is well-suited for modelling nitrate leaching due to its inherent interpretability, enabling stakeholders to understand the relationships among factors affecting leaching 108. It adeptly captures non-linear interactions in data, essential for the complex nature of nitrate leaching processes. Providing insights into variable importance, Reptree aids in identifying crucial factors influencing nitrate leaching 109. Its capability to handle multiple variables makes it suitable for this type of modelling, offering transparency and clarity in environmental decision-making. Response surface methodology (RSM).

The effects of three independent variables (clay (A, g), oil (B, ml), and age (C, days)) on nitrate leaching were optimised using RSM. The simplest model based on a first-order polynomial and quadratic model which can be used in RSM are introduced with the following Eq. (1) and Eq. (2), respectively

1 $${\text{y}}={\beta }_{0}+{\sum }_{i}^{k}{\beta }_{i}{x}_{i}+\upbeta$$

2 $${\text{y}}={\beta }_{0}+{\sum }_{i}^{k}{\beta }_{i}{x}_{i}+{\sum }_{i=1}^{k}\sum_{j\ge i}^{k}{\beta }_{ij }{x}_{i }{x}_{j}+\beta$$

where ${\beta }_{0}$ is the constant, ${\beta }_{i}$ is the linear coefficient and ${\beta }_{ij}$ interactive coefficient, i and j are the linear and quadratic coefficient, respectively. β is random test error, k is the number of factors, y is the estimated response, ${x}_{i}$ and ${x}_{j}$ are independent factors.

The central composite design (CCD) was used. The list the levels for the CCD and their coded values. 20 combinations with three replicas at a central location made up the entire design, which was carried out in a random order and has been presented in Table S1, S2 and S3 for T1, T2 and T3 type coating respectively in supplementary material. Design expert 13 software was used for the analysis.

### Statistical assessment and validation

Various statistical metrics of model correctness were calculated in addition to Taylor diagrams to assess and contrast the performances of the models. Nitrate leaching measurements and expected values were contrasted throughout the experiment. Statistical measures used to validate the Ml techniques include root mean square error (RMSE)^109,110^ which measures the average magnitude of the errors between predicted and observed values mean absolute error (MAE)^111,112^ which is the average of the absolute errors between predicted and observed values, Nash–Sutcliffe efficiency (NSE)^113–115^ which evaluates the efficiency of a model by comparing the simulated values to the observed values, relative to the mean observed value, Willmott index (WI)^116–118^ which assesses the agreement between observed and predicted values, considering both bias and variance, and correlation coefficient (r) which measures the linear correlation between predicted and observed values were used in statistical analysis to examine the effectiveness of the applied algorithms (i.e., ANN, SVM, M5P, RF, and REPTree). Additionally, graphical analysis was used to assess qualitative performance. The algorithm with the highest NSE, WI, and r values and the lowest MAE and RMSE values among the meta-heuristic algorithms were chosen to be the most accurate. The following are all the provided parameters: X_oi_ and X_pi_ are the ith observed and predicted values, respectively, and X̄_o_ and X̄_p_ are the mean observed and predicted values, respectively; n is the number of data points. Statistical metrics used in this study for evaluation, along with their formulae and ranges, are given in Table 2.

**Table 2:** Statistical parameters along with formulae and ranges used for the study.

Sl no	Name of the statistical parameters	Formula	Range
1	Root mean square error (RMSE)	$\sqrt{\frac{1}{n}. \sum_{i=1}^{n}{\left({{\text{x}}}_{{\text{pi}}}-{{\text{x}}}_{{\text{oi}}}\right)}^{2}}$	0 to ∞
2	Mean absolute error (MAE)	$\frac{1}{n} \sum_{i=1}^{n}\mid {{\text{x}}}_{{\text{pi}}}-{{\text{x}}}_{{\text{oi}}}\mid$	0 to ∞
3	Nash–Sutcliffe efficiency (NSE)	$1-[\frac{\sum_{i=1}^{n}{\left({{\text{x}}}_{{\text{pi}}}-{{\text{x}}}_{{\text{oi}}}\right)}^{2}}{\sum_{i=1}^{n}{\left({{\text{x}}}_{{\text{pi}}}-\overline{{{\text{x}}} }_{{\text{p}}}\right)}^{2}}]$	1 to −∞
4	Willmott index (WI)	$1 - \left[ {\frac{{\mathop \sum \nolimits_{i = 1}^{n} \left( {{\text{x}}_{{{\text{pi}}}} - {\text{x}}_{{{\text{oi}}}} } \right)^{2} }}{{\mathop \sum \nolimits_{i = 1}^{n} \left( {\left| {{\text{x}}_{{{\text{pi}}}} - {\overline{\text{x}}}_{{\text{p}}} } \right| + \left| {{\text{x}}_{{{\text{oi}}}} - {\overline{\text{x}}}_{{\text{o}}} } \right|} \right)^{2} }} } \right]$	0 to 1
5	Correlation coefficient (R)	$\frac{{\mathop \sum \nolimits_{i = 1}^{n} \left( {{\text{x}}_{{{\text{oi}}}} - {\overline{\text{x}}}_{{\text{o}}} } \right)\left( {{\text{x}}_{{{\text{pi}}}} - {\overline{\text{x}}}_{{\text{p}}} } \right){ }}}{{\sqrt {\mathop \sum \nolimits_{i = 1}^{n} \left( {{\text{x}}_{{{\text{oi}}}} - {\overline{\text{x}}}_{{\text{o}}} } \right)^{2} \mathop \sum \nolimits_{i = 1}^{n} \left( {{\text{x}}_{{{\text{pi}}}} - {\overline{\text{x}}}_{{\text{p}}} } \right)^{2} } }}$	+ 1 to − 1

### Ethics approval

All authors comply with the guidelines of the journal *Scientific Reports.*

### Consent to participate

All authors agreed to participate in this study.

## Results and discussion

### Effect of different proportions of coating material on nitrate leaching of coated USG

Sixteen compositions shown in Table 1 were coated and kept for 1–5 days (age) as a curing period, making the total number of treatments 80. They were put in the soil column, and nitrate leaching was calculated at an interval of 8 days for 32 days. The result has been presented in Table. S4, S5 and S6 for T1, T2, and T3, respectively in supplementary material. Analysis of variance (ANOVA) corresponding to ccd analysis, showing the effect of different proportions of coating material on nitrate leaching of three types of coating has been presented in Table 3.

**Table 3:** ANOVA for effect of coating material on nitrate leaching of 3 types of coating.

Sources	T1 coating		T2 coating		T3 coating	
	MS	F-val	MS	F-val	MS	F-val
A	0.09	7.34****	0.13	3.71^NS^*	0.54	16.89***
B	0.20	16.54***	0.08	2.26^NS^**	0.23	7.21****
C	0.39	32.65****	0.55	15.9**	0.20	6.38****
A*B	0.14	12.20^NS^**	0.01	0.41^NS^**	0.00	0.05^NS^**
A*C	0.02	1.69^NS^**	0.04	1.18^NS^**	0.11	3.41^NS^*
B*C	0.01	0.71^NS^**	0.03	0.73^NS^**	0.00	0.06^NS^**
A^2^	0.21	18.01***	0.02	0.62^NS^**	0.16	5.09****
B^2^	0.12	9.76****r	0.04	1.19^NS^**	0.17	5.28****
C^2^	1.09	92.14*	0.27	7.81****	0.79	24.88**
Model *P* value	< 0.0001		0.02		0.0024	
Model F value	19.75*		3.81****		7.20***	
R^2^	0.94		0.77		0.86	
Adj R^2^	0.89		0.57		0.74	
Lack of fit	3.13^NS^**		4.76^NS^**		4.87^NS^*	

*Significant at *p* < 0.0001; **Significant at *p* < 0.001; ***Significant at *p* < 0.01; ****Significant at *P* < 0.05; NS* nonsignificant at *P* > 0.05; NS**, nonsignificant at *P*>0.1; Adj. R2, adjusted R2. NS is the abbreviation to represent non-significant effects, A = Neem oil/Acacia oil, B = Bentonite clay/sulfur, c = Age.

### Effect of input coating parameters on nitrate leaching of T1 type coating

**Figure 4:**
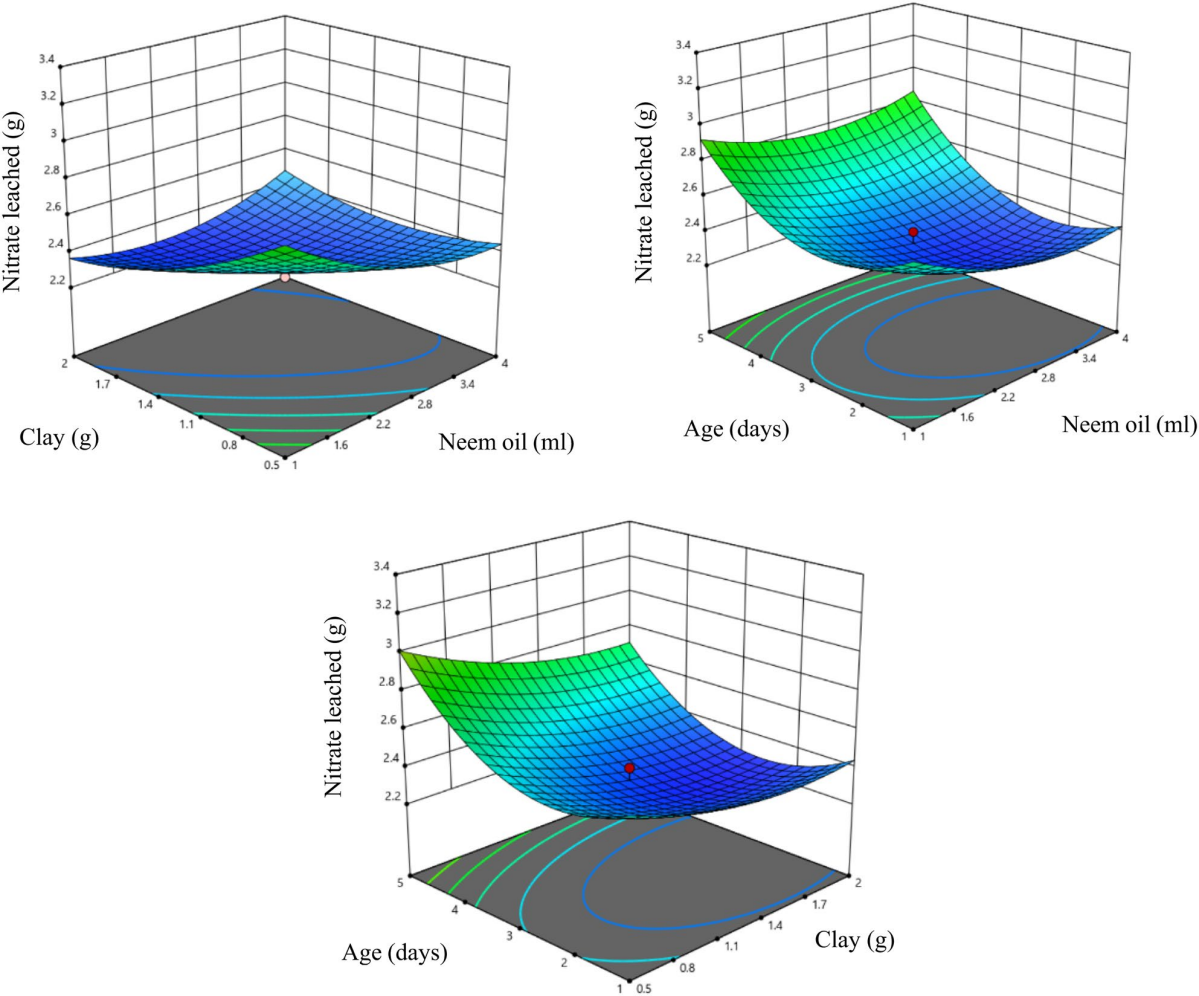
Response surface of nitrate leaching with T1 coating.

Figure 4 shows the response surface of nitrate leaching when USG was coated with T1 type coating. As mentioned in Table 3 bentonite clay has highly significant (*P* < 0.01) effect on nitrate leaching which shows that by increasing the clay content, Nitrate leaching can be reduced. Oil and age also have significant (*P* < 0.05) effect of nitrate leaching which means by changing the amount of oil content and curing period, nitrate leaching changed. As shown in Fig. 4, leaching decrease as clay content is increased. As clay content increases, the coating on fertilizer granules becomes thicker. This thicker coating reduces the rate at which nutrients can diffuse out of the granules into the soil. Consequently, nutrient release is slower in soils with higher clay content, affecting the availability of nutrients to plants. It is also supported by the study conducted by^119^. In the study by^120^, the effect of increasing oil content on nitrate leaching was observed. Initially, nitrate leaching decreased with higher oil content, possibly because neem oil acted as a binder, effectively adhering clay to Urea Super Granules (USGs). This binding reduced nutrient release and leaching. However, with excessive neem oil, the coating may become overly runny and weak, compromising its ability to control nutrient release. This could explain the later increase in nitrate leaching. Therefore, finding the right balance in oil content is crucial to optimize the effectiveness of USGs in nutrient management while minimizing environmental impacts. The observed phenomenon can be attributed to the progressive setting of the coating on Urea Super Granules (USGs) over time. As the coating matures beyond a certain period (typically more than three days), it achieves a uniform thickness, as supported by^19,54^. However, as the coating material dries up with increasing age, cracks may develop, allowing water to penetrate. This can lead to a sudden and catastrophic release of nutrients from the granules. Hence, the timing of coating maturity is critical, as it influences the integrity of the protective layer and the controlled release of nutrients, ultimately impacting nutrient management in agriculture. .

### Response surface of nitrate leaching in T2 type coating

**Figure 5:**
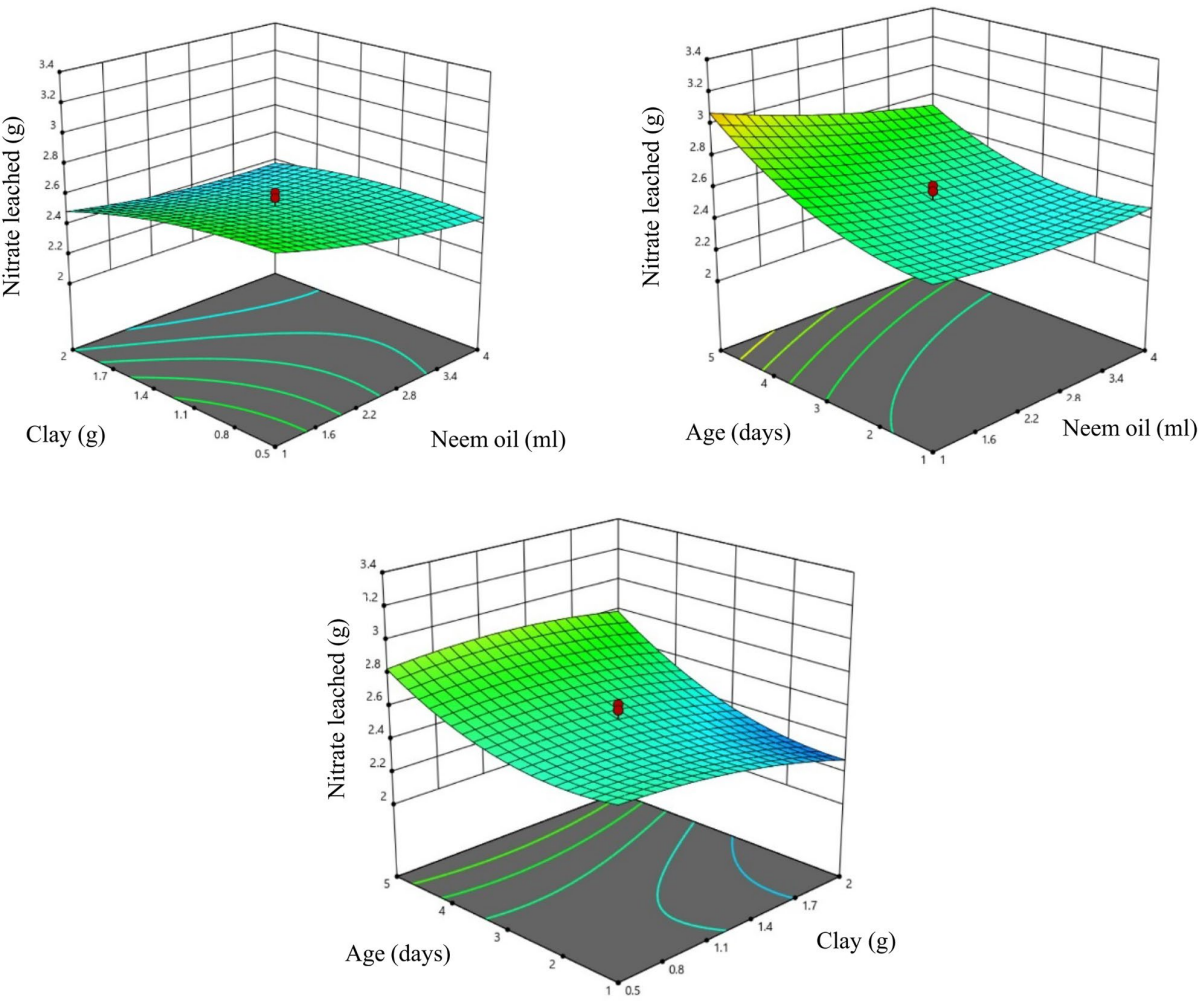
Response surface of nitrate leaching with T2 coating.

In the case of T2, as mentioned in Table 3, only age has the significant effect (*P* < 0.05) on nitrate leaching, while other two factors don’t have any significant effect. Which shows that nitrate leaching can be varied by changing curing period only. Figure 5 shows heating clay before coating, minimum nutrient leaching occurs when the age was one day and increases after that. It might be due to the fact given by^121^. that the application of heat to clay can lead to an increase in its surface area. This occurs because heat drives the expulsion of water and organic matter from the clay structure, causing it to expand and create more surface area. When such clay is used as a coating on Urea Super Granules (USGs), the increased surface area can result in a thicker coating. This is because the clay particles, now more exposed, can bond together more densely, forming a thicker and more protective layer around the USGs. A thicker coating can, in turn, impact the rate and uniformity of nutrient release from the granules in agricultural applications.

### Response surface of nitrate leaching in T3 type coating

**Figure 6:**
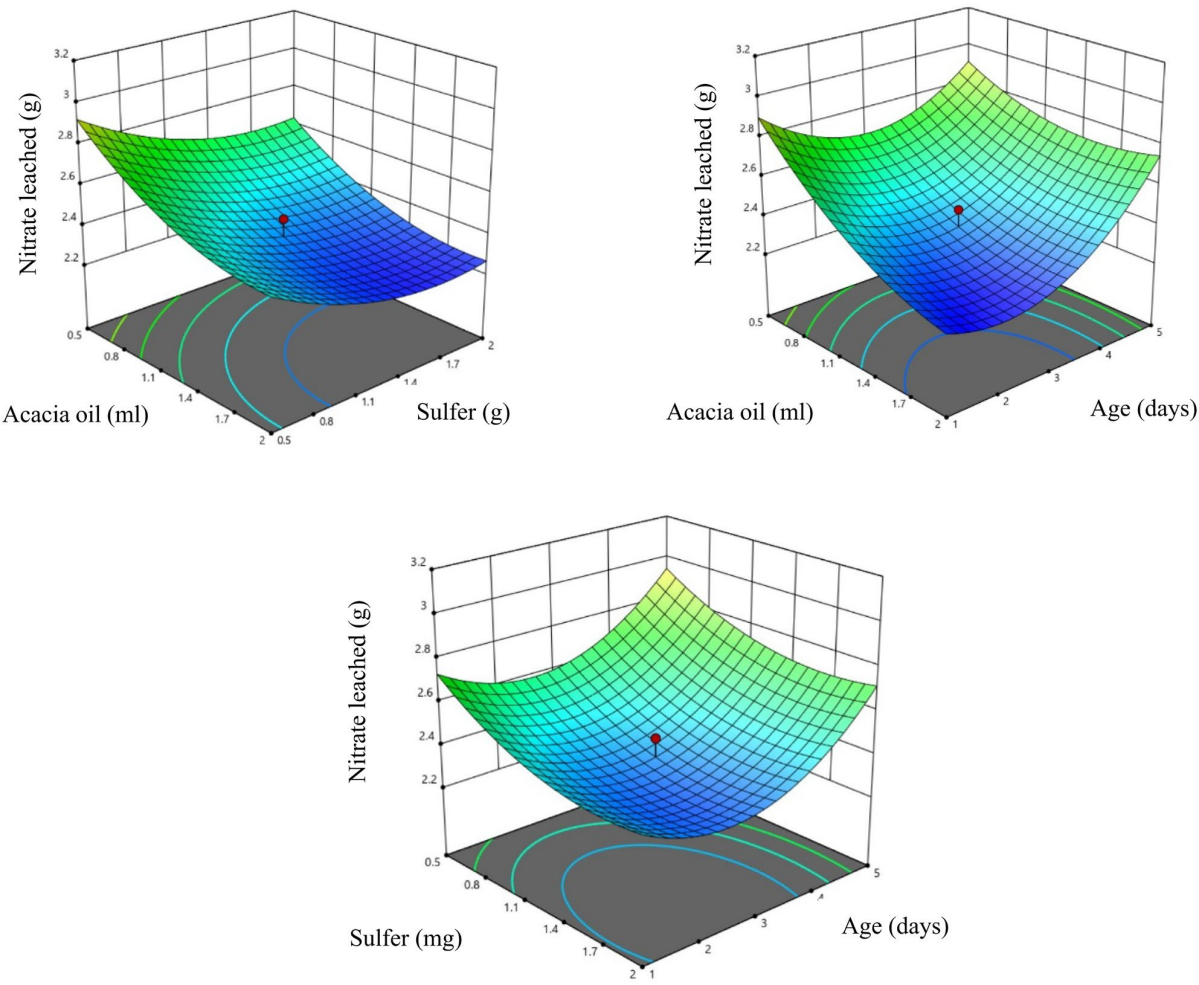
Response surface of nitrate leaching with T3 coating.

From Table 3., it was observed that acacia oil affects nitrate leaching significantly at 1% level (*P* < 0.01), whereas sulfur and age affect nitrate leaching at 5% level. It shows that nitrate leaching can be varied by varying any of the parameters. By increasing the acacia oil content, nitrate leaching decreased and was lowest at the oil content of 1.7 ml. It was observed that with increase in curing period, nitrate leaching first decreased and then increased with the lowest being observed at 1.6 days. In the case of T3 coating, it was observed that although the release of nutrients was slow for the initial days increasing thereon (Fig. 6), it was higher than both heating and non-heating type clay coating. It might be due to the fact that sulfer is a poor coating material^122^.

### Model validation

The entire data set was divided into 2 data sets named training and testing datasets. The training data set contains 80% of the data, and the testing data set contains 20%. The nitrate leaching of nine compositions was estimated using machine learning techniques (i.e., ANN, SVM, M5P, RF, REPTree and RSM).

#### Modelling of nitrate leaching of T1 type coating

The performance of applied algorithms was assessed by employing performance indicators. Performance indicators of different ML algorithms of testing datasets are presented in Table 4. The scatter plot of the testing data set has been presented in Fig. F1 in the supplementary material.

**Table 4:** Performance indices for meta‐heuristic algorithms‐based models during the testing phase.

Applied models	Testing				
	R	MAE	RMSE	NSE	WI
ANN	0.55	0.20	0.25	0.29	0.71
SVM	0.56	0.21	0.25	0.30	0.70
M5P	0.75	0.16	0.20	0.55	0.82
RF	0.86	0.13	0.16	0.72	0.90
REPTree	0.72	0.17	0.20	0.52	0.82
RSM	0.97	0.07	0.08	0.94	0.99

**Figure 7:**
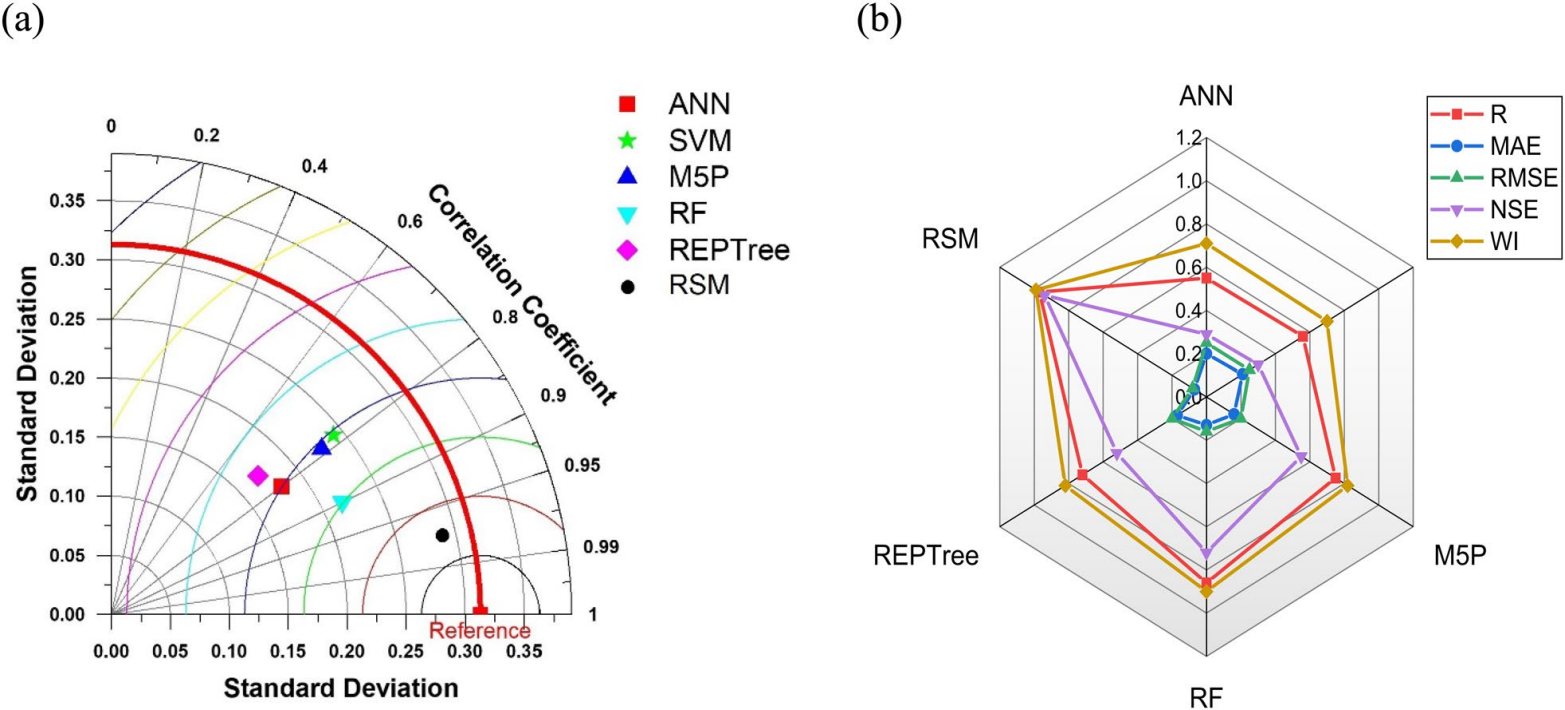
(**a**) Taylor diagram and (**b**) Radar chart representing the efficiency of different applied ML algorithms of type 1 coating in the testing phase.

RSM model showed the best performance, followed by RF, M5P and REPTree. The least accurate model was ANN, followed by SVM. The observed performance of the Artificial Neural Network (ANN) model, excelling in training but demonstrating limited generalization ability, is likely due to the dataset's small size comprising only 60 training samples. This insufficiency can lead to overfitting, where the model becomes overly tailored to the training data and struggles to make accurate predictions on new data^123^. Study aligned with this perspective, emphasizing the significance of dataset size in model performance. To enhance the ANN's generalization capacity, acquiring a more extensive and diverse dataset is essential. This allows the model to better understand underlying patterns, resulting in improved performance on unseen data and more reliable predictions . On the other hand, RSM performed the best among all the models. The superior performance of Response Surface Methodology (RSM) often arises from its appropriateness for well-understood models with a limited number of variables. RSM is particularly effective in optimizing processes or systems where the relationships between variables are relatively clear, enabling it to efficiently pinpoint optimal conditions and achieve maximum outcomes with fewer experimental trials.As in this study only 3 input variables and one output are there, this may be very well be the cause of better performance of RSM. The enhanced performance of Random Forest (RF) is widely documented in literature and can be attributed to its unique capabilities. RF not only assesses the influence of predictors on the predictand but also quantifies the importance of each predictor in predicting the outcome^124^. This dual approach distinguishes RF from many other modelling techniques. By assigning importance scores to predictors, RF provides valuable insights into the relative contribution of each variable to the model's accuracy. This information aids in feature selection, model interpretation, and the overall optimization of predictive performance, making RF a powerful and versatile tool in various fields, including machine learning and data analysis. The performance comparison of different algorithms has been presented by the Taylor diagram and radar chart as presented in Fig. 7a and b respectively.

#### Estimation of nitrate leaching of T2 type coating

**Figure 8:**
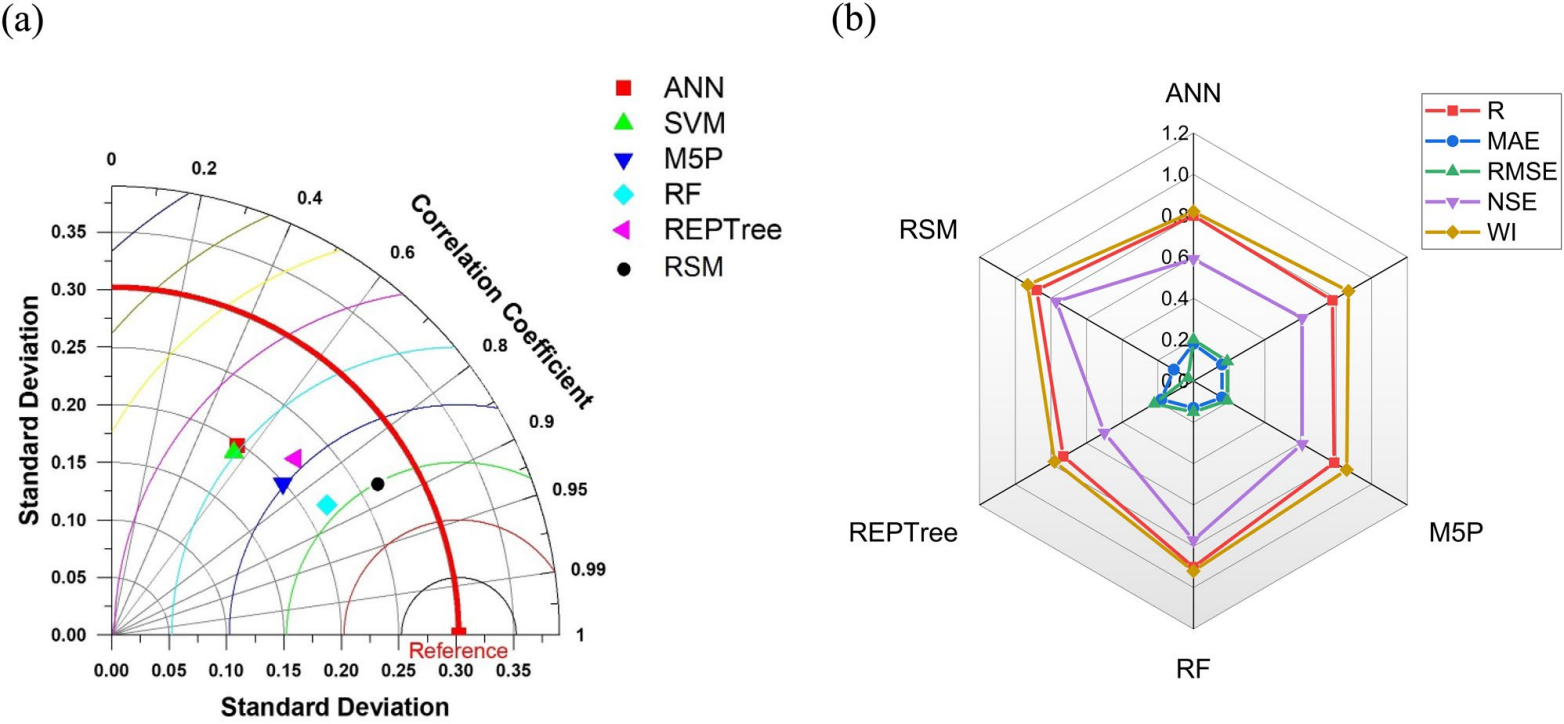
(**a**) Taylor diagram and (**b**) Radar chart representing the efficiency of different applied ML algorithms of type 2 coating in the testing phase.

Utilising the same performance indicators with the coated USG, the effectiveness of the applied ML algorithms was evaluated and is shown in Table 5. The scatter plot of the testing data set has been presented in Fig. F2 in the supplementary material. As presented in Table 4 and confirmed by Fig. 10, RSM was the best fitting model followed by RF like in the T1 coating type case. For efficient performance of ML algorithms huge amount of dataset is required^125^ i.e. more the data available, better is the performance of ML algorithms. In this study as the number of datasets is less (80 dataset), this may be the cause of subpar performance of ML algorithms. Taylor diagram and radar chart to compare the efficiency of different algorithms have been presented in Fig. 8a and b, respectively. The ranking of different algorithms was done according to the percentage error and has been presented in Fig. 10.

**Table 5:** Performance indices for meta‐heuristic algorithms‐based models during the testing phase.

Machine learning algorithms	Testing				
	R	MAE	RMSE	NSE	WI
ANN	0.80	0.18	0.20	0.59	0.82
SVM	0.78	0.16	0.19	0.61	0.87
M5P	0.79	0.16	0.19	0.61	0.86
RF	0.90	0.13	0.15	0.77	0.92
REPTree	0.73	0.18	0.22	0.50	0.78
RSM	0.88	0.11	0.03	0.77	0.93

#### Estimation of nitrate leaching of T3 type coating

The performance indicators of the algorithms used to predict nitrate leaching from sulfer-coated USG have been presented in Table 6. Fig. F3 in the supplementary material shows the scatter plot of the testing data set. It has been observed that RSM outperformed other models followed by SVM.

**Table 6:** Performance indices for meta‐heuristic algorithms‐based models during the testing phase.

Machine learning algorithms	Testing				
	R	MAE	RMSE	NSE	WI
ANN	0.86	0.11	0.13	0.73	0.92
SVM	0.87	0.10	0.12	0.75	0.93
M5P	0.85	0.10	0.13	0.72	0.91
RF	0.84	0.11	0.13	0.69	0.90
REPTree	0.85	0.11	0.13	0.71	0.91
RSM	0.93	0.11	0.13	0.87	0.96

scatter plot of the testing data set representing observed and model-predicted data has been presented in Fig. F3 in the supplementary material. Additional confirmation of the superior performance of RSM was also done by the Taylor diagram and radar chart, as presented in Fig. 9a and b, respectively.

**Figure 9:**
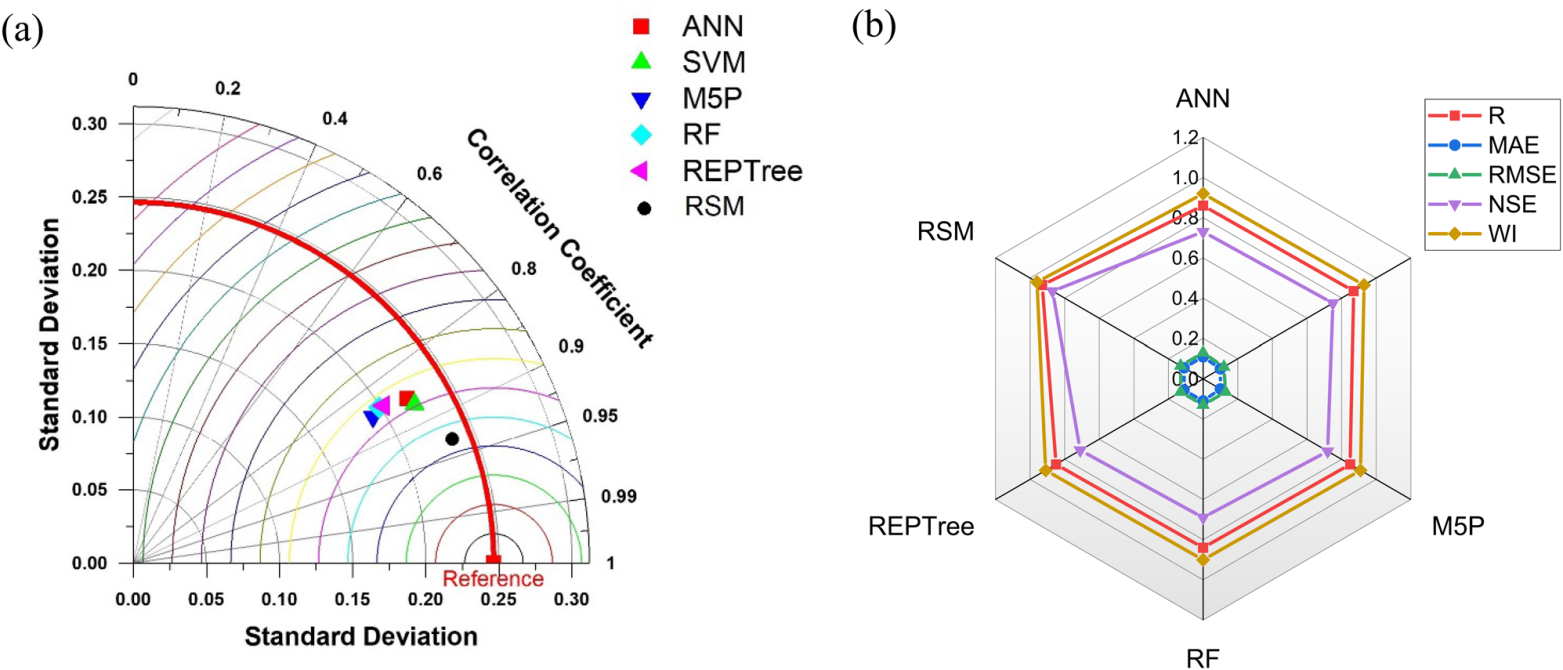
(**a**) Taylor diagram and (**b**) Radar chart representing the efficiency of different applied ML algorithms of type 3 coating in the testing phase.

It was also observed that there was no significant difference between the five algorithms (i.e. ANN, SVM, M5P, RF and REPTree) while predicting the nitrate leaching of T3 coating. Efficient prediction of RSM has been discussed earlier. The better performance of SVM was also supported by a previous study presented by^36^ who stated that superior performance of SVM might be due to the fact that it can forecast environmentally stable isotopes indirectly, quickly, and conveniently by precisely simulating NO_3_ concentrations in surface water using widely measured hydro-chemical variables.

### Optimality of coating material

Response surface method (RSM) was used as an optimization technique to find the best composition ratio of input variables. Achieving the lowest nitrate leaching after 32 days is the goal of optimization. Each response's significance level is provided with equal weight. The experimental data is chosen to represent the lowest and highest values. Constraints for optimization in RSM was taken as minimization of Nitrate leaching. Experiments were conducted on the model predicted optimized value. Model predicted values, observed values along with deviation has been presented in Table 7.

**Table 7:** Optimum value of parameters with deviation for three types of coating.

Coating type	Optimized Input parameters			Nitrate leaching		Deviation (%)
	Neem/acacia oil (gm)	Bentonite clay /sulfer (ml)	Age	Predicted	Observed	
T1	2.61	1.67	2.44	2.26	2.28	0.8
T2	2.18	2	1.01	2.27	2.26	0.4
T3	1.69	1.64	2.18	2.20	2.22	0.9

### Model comparison

**Figure 10:**
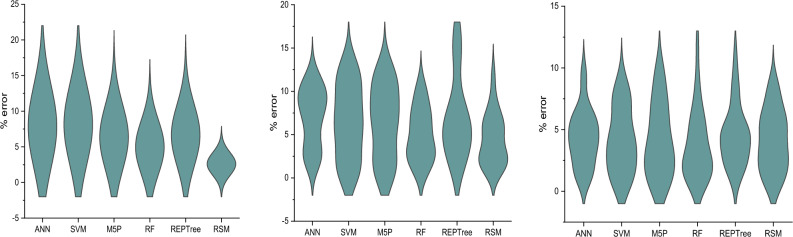
Error comparison of different ml algorithms for T1 to T3 (left to right) types of coating.

The distribution and the extreme value of percentage error have been presented in Fig. 10.

RSM performed well in all three types of coating. It may be due to the fact that (1) RSM performs well when dealing with limited number of variables^126^ (2) RSM is an efficient model than ML when the dataset is small and no of variables are limited^125^. As in this study only 80 datasets are used, this may be the cause of poor performance of ML algorithms compared to RSM. It was observed that RF has good result in all types of coating. Good performance of RF might be due to its advantages like (1) It’s resistance to overfitting^127,128^; (2) It’s user-friendly nature as it can work efficiently even with only two parameters and RF is typically not very sensitive to their values^129^; and (4) It is resistant to outliers which may be the principal cause in this study^130,131^. This interpretation is also in accordance with^129^, who state that the better performance of RF is due to its ability to handle nonlinear relationships between the nitrate leaching and predictor variables. In case of T3 type coating SVM performed well, which is in line of the previous studies done by (1)^132^, who stated that SVM could generate satisfactory accuracy with smaller size of training dataset; (2) It depends on fewer datapoints to decide the position of decision surface^133,134^.

### Comparision of this study with previous studies

**Figure 11:**
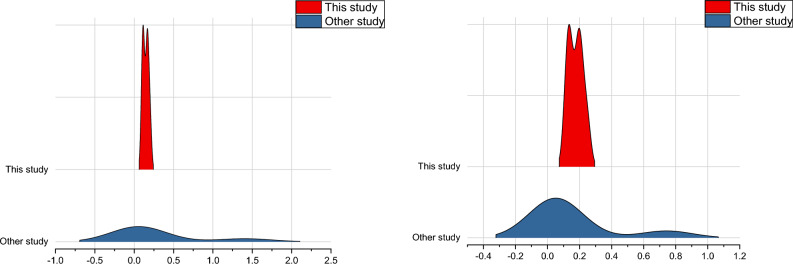
Ridgeline plot of MAE (left) and RMSE (Right) of this study in comparison to past studies.

It was found that RSM, ANN and RF were the best models for each type of coating. The result of this study was compared with past studies. Mean absolute error (MAE) and root mean squared error (RMSE) were taken as the statistical parameters to compare the result of this study with previous studies. The parameters taken in this study and their relationship in the leaching were an improvement over other past study. This study outperformed the result given by^135^, who applied eight different machine learning algorithms for the prediction of the water quality index (WQI) of groundwater and found the best fitting algorithm was multilinear regression with statistical parameters of MAE and RMSE to be 1.45 and 2.14 respectively. The application of ML algorithms for leaching other than nitrate is also compared. Zhang et al. (2022) used ML algorithms in hydrometallurgy and found the best-fit model (SVM) has an RMSE value of 5.004, respectively^136^. The ridgeline plot comparing the result of this study with the past studies has been presented in Fig. 11 below.

### Limitations and future directions

The current study examined the ability of six metaheuristic algorithms (viz., SVM, ANN, RF, M5P, REPTree, and RSM) to predict and optimize nitrate leaching from USGs using different binding material and binding agent and curing period of coating as the dominant predictor variables. Some of the algorithms used in the study were found very efficient in predicting nitrate leaching. However, the efficacy of these models could be improved using advanced optimisation algorithms such as genetic algorithm and particle swarm optimisation. Future studies could consider some predictors influenced by feedback loops (e.g., nutrient cycling, soil organic matter, water movement feedback) that significantly affect the nitrate leaching process to reduce the prediction uncertainty. Subsequent studies could also test different coating materials such as biopolymers, organic materials, or some synthetic materials to enhance the effectiveness of nitrate leaching and reduce the cost.

## Conclusion

USG was coated with three types of coating, viz. bentonite clay & neem oil without application of heat, bentonite clay & neem oil with the application of heat and sulfer & acacia oil. 10 gm of each type of coated USG was put in soil columns containing 750 gm of soil, followed by 250 gm of soil. All the columns are irrigated up to the saturation. The leachate of each soil column was collected in the container placed below at eight days intervals for 32 days. The nitrate content of the leachate was found by using the cd reduction column method. The data obtained were evaluated by potential meta-heuristic approaches in forecasting nitrate leaching of USG, viz., artificial neural networks (ANN), support vector machines (SVM), M5 model trees (M5P), random forests (RF), reduced error pruning trees (REPTree) and Response surface methodology (RSM). Using well-known performance metrics (such as RMSE, MAE, NSE, WI, and r). the following outcomes were observed from this study. Optimization of coating parameters were done for minimizing the nitrate leaching.

1. USG with the coating is an efficient method of application of fertiliser with slow-release characteristics.
2. Response surface methodology was the best predictive model for all types of coating. Random forest and support vector machine can be used to model nitrate leaching for USG.
3. Neem oil/ Acacia oil (ml): clay/sulfur (g) : age (days) for minimium nitrate leaching was found to be 2.61: 1.67: 2.4 for T1, 2.18: 2: 1 for T2 and 1.69: 1.64: 2.18 for T3.
4. When USG is coated with bentonite and neem oil, it should be kept for three days as curing time. If time is a constraint, bentonite clay can be heated, which can make fertiliser ready in 1 day.

Optimizing the coating proportions will enable farmers to use fewer fertilizers, thereby increasing their income. Additionally, reduced leaching will contribute to a decrease in groundwater pollution. Overall, the methodology created allows for the prediction of nitrate leaching using a model trained on the observed data as input, which could be a useful tool for agronomists, soil scientists, and environmentalists to ensure the most effective application of fertiliser and the sustainable management of available resources.

### Supplementary Information

Supplementary Information.

## Data Availability

The datasets used and/or analysed during the current study available from the corresponding author on reasonable request.
