# Supplementary material for: Proportional impact prediction model of coating material on nitrate leaching of slow-release Urea Super Granules (USG) using machine learning and RSM technique
Source: Sci Rep. 2024 Feb 6;14:3053. doi: 10.1038/s41598-024-53410-8 (PMC10847469; doi:10.1038/s41598-024-53410-8)
Supplement: Supplementary file 1 — Supplementary Information. [file 41598_2024_53410_MOESM1_ESM.docx]

Table S1 ccd design for T1 type coating

| Run no | Neem oil (ml) | Clay (mg) | Age (days) | Nitrate leaching (gm) |
| --- | --- | --- | --- | --- |
| 1 | 1 | 2 | 1 | 2.50903 |
| 2 | 4 | 0.5 | 1 | 2.31899 |
| 3 | 2.5 | 1.25 | 3 | 2.258 |
| 4 | 1 | 0.5 | 1 | 2.97171 |
| 5 | 4 | 2 | 5 | 2.9206 |
| 6 | 5.02269 | 1.25 | 3 | 2.5748 |
| 7 | 2.5 | 1.25 | 3 | 2.396 |
| 8 | 4 | 0.5 | 5 | 2.9761 |
| 9 | 2.5 | 1.25 | 3 | 2.258 |
| 10 | 2.5 | 1.25 | 6.36359 | 3.371 |
| 11 | 2.5 | 1.25 | -0.363586 | 2.897 |
| 12 | 2.5 | 1.25 | 3 | 2.396 |
| 13 | 2.5 | -0.0113446 | 3 | 2.9275 |
| 14 | 2.5 | 2.51134 | 3 | 2.2921 |
| 15 | 4 | 2 | 1 | 2.62903 |
| 16 | 2.5 | 1.25 | 3 | 2.258 |
| 17 | 1 | 2 | 5 | 2.8366 |
| 18 | 2.5 | 1.25 | 3 | 2.396 |
| 19 | 1 | 0.5 | 5 | 3.193 |
| 20 | -0.0226892 | 1.25 | 3 | 2.826 |

Table S2 ccd design for T2 type coating

| Run no | Neem oil (ml) | Clay (mg) | Age (days) | Nitrate leaching (gm) |
| --- | --- | --- | --- | --- |
| 1 | 2.5 | 1.25 | 3 | 2.583 |
| 2 | 2.5 | 1.25 | -0.363586 | 2.738 |
| 3 | 2.5 | 1.25 | 3 | 2.381 |
| 4 | 2.5 | 2.51134 | 3 | 2.273 |
| 5 | 4 | 2 | 1 | 2.138 |
| 6 | 1 | 2 | 5 | 2.794 |
| 7 | 2.5 | 1.25 | 3 | 2.583 |
| 8 | 2.5 | 1.25 | 3 | 2.381 |
| 9 | 2.5 | 1.25 | 6.36359 | 3.286 |
| 10 | 4 | 0.5 | 1 | 2.54 |
| 11 | 2.5 | 1.25 | 3 | 2.583 |
| 12 | 1 | 2 | 1 | 2.364 |
| 13 | 5.02269 | 1.25 | 3 | 2.543 |
| 14 | 2.5 | -0.0113446 | 3 | 2.671 |
| 15 | 4 | 2 | 5 | 2.846 |
| 16 | 4 | 0.5 | 5 | 2.458 |
| 17 | -0.0226892 | 1.25 | 3 | 2.924 |
| 18 | 1 | 0.5 | 1 | 2.37 |
| 19 | 1 | 0.5 | 5 | 3.14 |
| 20 | 2.5 | 1.25 | 3 | 2.619 |

Table S3 ccd design for T3 type coating

| Run | Neem oil (ml) | Clay (mg) | Age (days) | Nitrate leaching (gm) |
| --- | --- | --- | --- | --- |
| 1 | 2 | 2 | 1 | 2.238 |
| 2 | 2 | 0.5 | 5 | 2.9173 |
| 3 | 2 | 0.5 | 1 | 2.3682 |
| 4 | 1.25 | 1.25 | -0.363586 | 3.042 |
| 5 | 1.25 | 1.25 | 3 | 2.454 |
| 6 | 1.25 | 1.25 | 3 | 2.246 |
| 7 | 2 | 2 | 5 | 2.8845 |
| 8 | 1.25 | 1.25 | 3 | 2.454 |
| 9 | 1.25 | -0.0113446 | 3 | 3.143 |
| 10 | 1.25 | 1.25 | 3 | 2.246 |
| 11 | 0.5 | 2 | 1 | 2.7831 |
| 12 | 1.25 | 1.25 | 3 | 2.454 |
| 13 | 1.25 | 2.51134 | 3 | 2.348 |
| 14 | 1.25 | 1.25 | 6.36359 | 3.164 |
| 15 | -0.0113446 | 1.25 | 3 | 3.164 |
| 16 | 0.5 | 0.5 | 1 | 2.9305 |
| 17 | 2.51134 | 1.25 | 3 | 2.316 |
| 18 | 0.5 | 0.5 | 5 | 3.0504 |
| 19 | 1.25 | 1.25 | 3 | 2.318 |
| 20 | 0.5 | 2 | 5 | 2.9274 |

Table S4 Nitrate leaching of different composition of USG with T1 coating (a) age is 1 day (b) age is 2 days (c) age is 3 days (d) age is 4 days (e) age is 5 days

| composition | Duration | | | | |
| --- | --- | --- | --- | --- | --- |
|  | 8 days | 16 days | 24 days | 32 days | Total |
| 1 | 1193.912 | 1389.213 | 254.08 | 134.507 | 2971.712 |
| 2 | 1151.562 | 1261.665 | 246.424 | 100.961 | 2760.612 |
| 3 | 1011.076 | 1002.655 | 174.967 | 119.557 | 2308.255 |
| 4 | 1048.554 | 994.554 | 219.509 | 56.371 | 2318.988 |
| 5 | 1000.306 | 1089.199 | 462.592 | 69.808 | 2621.905 |
| 6 | 1057.918 | 1176.161 | 334.467 | 96.419 | 2664.965 |
| 7 | 897.416 | 1109.628 | 531.23 | 206.857 | 2745.131 |
| 8 | 1104.248 | 929.007 | 207.219 | 47.144 | 2287.618 |
| 9 | 940.809 | 1054.783 | 373.122 | 103.504 | 2472.218 |
| 10 | 855.998 | 939.84 | 498.2 | 54.642 | 2348.68 |
| 11 | 823.159 | 880.001 | 437.889 | 39.372 | 2180.421 |
| 12 | 1171.562 | 800.338 | 515.347 | 190.839 | 2678.086 |
| 13 | 1274.131 | 724.034 | 385.986 | 124.878 | 2509.029 |
| 14 | 1146.586 | 685.586 | 495.249 | 173.93 | 2501.351 |
| 15 | 1050.974 | 738.956 | 360.556 | 203.937 | 2354.423 |
| 16 | 1276.431 | 696.948 | 470.613 | 185.04 | 2629.032 |

(a)

| composition | Duration | | | | |
| --- | --- | --- | --- | --- | --- |
|  | 8 days | 16 days | 24 days | 32 days | Total |
| 1 | 1067.636 | 1150.609 | 260.476 | 192.513 | 2671.234 |
| 2 | 1033.998 | 1099.476 | 321.876 | 189.638 | 2644.988 |
| 3 | 992.392 | 1045.865 | 390.551 | 186.346 | 2615.154 |
| 4 | 1158.08 | 930.242 | 333.741 | 139.024 | 2561.087 |
| 5 | 905.883 | 958.247 | 472.673 | 121.476 | 2458.279 |
| 6 | 952.915 | 1009.94 | 332.433 | 153.946 | 2449.234 |
| 7 | 779.394 | 864.032 | 470.048 | 109.601 | 2223.075 |
| 8 | 1090.774 | 892.797 | 412.075 | 110.583 | 2506.229 |
| 9 | 886.736 | 913.587 | 378.122 | 161.01 | 2339.455 |
| 10 | 768.16 | 827.688 | 524.711 | 78.815 | 2199.374 |
| 11 | 735.852 | 786.721 | 500.609 | 124.8 | 2147.982 |
| 12 | 1084.84 | 738.913 | 468.377 | 174.54 | 2466.67 |
| 13 | 1224.077 | 794.483 | 307.63 | 123.147 | 2449.337 |
| 14 | 1074.777 | 853.746 | 382.677 | 64.449 | 2375.649 |
| 15 | 997.644 | 804.678 | 336.34 | 166.266 | 2304.928 |
| 16 | 1064.661 | 887.679 | 333.721 | 153.46 | 2439.521 |

(b)

| composition | Duration | | | | |
| --- | --- | --- | --- | --- | --- |
|  | 8 days | 16 days | 24 days | 32 days | Total |
| 1 | 1002.441 | 1088.032 | 241.41 | 104.576 | 2436.459 |
| 2 | 959.546 | 1054.739 | 257.192 | 71.226 | 2342.703 |
| 3 | 928.386 | 1029.353 | 352.153 | 135.939 | 2445.831 |
| 4 | 1074.667 | 932.71 | 318.126 | 76.595 | 2402.098 |
| 5 | 853.607 | 921.085 | 494.433 | 235.887 | 2505.012 |
| 6 | 886.119 | 963.614 | 371.862 | 89.531 | 2311.126 |
| 7 | 796.175 | 855.682 | 497.219 | 39.287 | 2188.363 |
| 8 | 1093.967 | 907.583 | 309.477 | 166.446 | 2477.473 |
| 9 | 827.679 | 903.95 | 433.961 | 150.47 | 2316.06 |
| 10 | 730.253 | 833.956 | 550.802 | 62.987 | 2177.998 |
| 11 | 692.461 | 793.691 | 585.001 | 32.548 | 2103.701 |
| 12 | 1066.846 | 913.641 | 506.817 | 33.829 | 2521.133 |
| 13 | 1064.07 | 836.592 | 281.707 | 144.301 | 2326.67 |
| 14 | 963.749 | 815.947 | 312.044 | 133.675 | 2225.415 |
| 15 | 891.397 | 934.74 | 218.989 | 133.46 | 2178.586 |
| 16 | 1014.846 | 867.246 | 358.31 | 118.24 | 2358.642 |

(c)

| composition | Duration | | | | |
| --- | --- | --- | --- | --- | --- |
|  | 8 days | 16 days | 24 days | 32 days | Total |
| 1 | 1149.988 | 1270.837 | 274.783 | 176.26 | 2871.868 |
| 2 | 1107.975 | 1221.397 | 420.124 | 275.486 | 3024.982 |
| 3 | 1056.51 | 1192.022 | 499.545 | 151.234 | 2899.311 |
| 4 | 1038.216 | 797.537 | 346.333 | 244.893 | 2426.979 |
| 5 | 985.51 | 1099.853 | 466.956 | 255.382 | 2807.701 |
| 6 | 1028.606 | 1152.276 | 425.676 | 163.739 | 2770.297 |
| 7 | 910.346 | 991.042 | 587.209 | 279.889 | 2768.486 |
| 8 | 1075.035 | 886.796 | 302.042 | 159.071 | 2422.944 |
| 9 | 916.77 | 1038.73 | 581.676 | 219.152 | 2756.328 |
| 10 | 844.541 | 952.744 | 547.821 | 180.768 | 2525.874 |
| 11 | 799.286 | 931.597 | 603.895 | 42.597 | 2377.375 |
| 12 | 1202.344 | 810.278 | 424.98 | 294.171 | 2731.773 |
| 13 | 1275.314 | 884.835 | 294.538 | 200.591 | 2655.278 |
| 14 | 1199.413 | 997.788 | 312.828 | 207.521 | 2717.55 |
| 15 | 1095.317 | 812.909 | 513.61 | 187.027 | 2608.863 |
| 16 | 1210.585 | 961.98 | 496.971 | 153.286 | 2822.822 |

(d)

| composition | Duration | | | | |
| --- | --- | --- | --- | --- | --- |
|  | 8 days | 16 days | 24 days | 32 days | Total |
| 1 | 1212.882 | 1346.974 | 371.439 | 262.012 | 3193.307 |
| 2 | 1189.583 | 1337.576 | 352.935 | 279.981 | 3160.075 |
| 3 | 1184.71 | 1312.882 | 341.624 | 283.919 | 3123.135 |
| 4 | 911.708 | 1029.293 | 423.393 | 243.424 | 2607.818 |
| 5 | 1106.493 | 1216.464 | 478.968 | 86.19 | 2888.115 |
| 6 | 1194.167 | 1280.684 | 412.854 | 95.859 | 2983.564 |
| 7 | 1032.487 | 1127.377 | 474.485 | 153.607 | 2787.956 |
| 8 | 1038.99 | 980.668 | 407.622 | 129.099 | 2556.379 |
| 9 | 1086.232 | 1167.573 | 344.567 | 229.002 | 2827.374 |
| 10 | 1026.992 | 1007.213 | 529.516 | 149.259 | 2712.98 |
| 11 | 927.057 | 896.365 | 585.115 | 218.815 | 2627.352 |
| 12 | 1200.821 | 957.121 | 411.595 | 186.444 | 2755.981 |
| 13 | 1186.914 | 1002.332 | 386.702 | 260.669 | 2836.617 |
| 14 | 1202.028 | 984.055 | 483.803 | 211.635 | 2881.521 |
| 15 | 1134.676 | 976.364 | 497.361 | 388.734 | 2997.135 |
| 16 | 1282.626 | 990.1471 | 431.4061 | 216.3701 | 2920.549 |

(e)

|  |  |
| --- | --- |
|  |  |
|  |  |

Fig F1. Scatter plot of the testing data set representing observed and model predicted data in T1 type coating

Table S5 Nitrate leaching of different composition of USG with T2 coating (a) age is 1 day (b) age is 2 days (c) age is 3 days (d) age is 4 days (e) age is 5 days

| composition | Duration | | | | |
| --- | --- | --- | --- | --- | --- |
|  | 8 days | 16 days | 24 days | 32 days | Total |
| 1 | 984.941 | 1067.108 | 229.207 | 88.961 | 2370.217 |
| 2 | 942.046 | 1036.375 | 247.549 | 55.611 | 2281.581 |
| 3 | 898.246 | 998.349 | 329.87 | 107.684 | 2334.149 |
| 4 | 1257.167 | 914.346 | 308.483 | 60.98 | 2540.976 |
| 5 | 836.107 | 902.721 | 481.143 | 220.272 | 2440.243 |
| 6 | 804.867 | 877.276 | 424.318 | 134.855 | 2241.316 |
| 7 | 778.675 | 837.318 | 487.576 | 23.672 | 2127.241 |
| 8 | 1176.467 | 876.549 | 287.164 | 138.161 | 2478.341 |
| 9 | 868.619 | 936.94 | 362.219 | 73.916 | 2241.694 |
| 10 | 704.41 | 807.249 | 532.816 | 39.029 | 2083.504 |
| 11 | 656.275 | 766.641 | 556.672 | 35.619 | 2015.207 |
| 12 | 1049.346 | 843.913 | 497.174 | 18.214 | 2408.647 |
| 13 | 1146.57 | 816.864 | 272.064 | 128.686 | 2364.184 |
| 14 | 946.249 | 778.649 | 283.467 | 99.126 | 2107.491 |
| 15 | 861.597 | 916.376 | 209.346 | 117.845 | 2105.164 |
| 16 | 997.346 | 797.518 | 348.667 | 102.625 | 2246.156 |

(a)

| composition | Duration | | | | |
| --- | --- | --- | --- | --- | --- |
|  | 8 days | 16 days | 24 days | 32 days | Total |
| 1 | 1053.376 | 1136.349 | 246.216 | 178.253 | 2614.194 |
| 2 | 1023.468 | 1088.946 | 311.346 | 179.108 | 2602.868 |
| 3 | 984.028 | 1037.501 | 382.187 | 177.982 | 2581.698 |
| 4 | 1142.146 | 814.308 | 317.807 | 123.09 | 2397.351 |
| 5 | 895.619 | 947.983 | 462.409 | 111.212 | 2417.223 |
| 6 | 874.819 | 901.67 | 366.205 | 149.093 | 2291.787 |
| 7 | 771.781 | 856.419 | 462.435 | 101.988 | 2192.623 |
| 8 | 1076.137 | 738.16 | 397.438 | 95.946 | 2307.681 |
| 9 | 939.372 | 996.397 | 318.89 | 140.403 | 2395.062 |
| 10 | 755.647 | 815.175 | 512.198 | 66.302 | 2149.322 |
| 11 | 723.492 | 774.361 | 488.249 | 112.44 | 2098.542 |
| 12 | 975.134 | 619.207 | 458.671 | 164.834 | 2217.846 |
| 13 | 1214.761 | 785.167 | 298.314 | 113.831 | 2412.073 |
| 14 | 1067.164 | 846.133 | 375.064 | 56.836 | 2345.197 |
| 15 | 986.138 | 793.172 | 324.834 | 154.76 | 2258.904 |
| 16 | 958.346 | 751.364 | 327.406 | 147.145 | 2184.261 |

(b)

| composition | Duration | | | | |
| --- | --- | --- | --- | --- | --- |
|  | 8 days | 16 days | 24 days | 32 days | Total |
| 1 | 1176.048 | 1371.349 | 236.216 | 116.643 | 2900.256 |
| 2 | 1126.246 | 1236.349 | 221.108 | 75.645 | 2659.348 |
| 3 | 998.716 | 987.295 | 159.607 | 104.197 | 2249.815 |
| 4 | 915.394 | 861.394 | 286.349 | 123.211 | 2186.348 |
| 5 | 988.956 | 1077.849 | 451.242 | 58.458 | 2576.505 |
| 6 | 922.193 | 1036.167 | 354.506 | 84.888 | 2397.754 |
| 7 | 875.703 | 1087.915 | 509.517 | 185.144 | 2658.279 |
| 8 | 994.605 | 819.364 | 297.576 | 137.501 | 2249.046 |
| 9 | 1026.264 | 1144.507 | 302.813 | 64.765 | 2538.349 |
| 10 | 842.407 | 926.249 | 484.609 | 41.051 | 2178.64 |
| 11 | 817.519 | 874.361 | 432.249 | 33.732 | 2157.861 |
| 12 | 1148.049 | 776.825 | 491.834 | 167.326 | 2584.034 |
| 13 | 1268.461 | 718.364 | 380.316 | 119.208 | 2486.349 |
| 14 | 1142.916 | 681.916 | 491.579 | 170.26 | 2486.671 |
| 15 | 1041.364 | 729.346 | 350.946 | 194.327 | 2315.983 |
| 16 | 1273.864 | 694.381 | 468.046 | 182.473 | 2618.764 |

(c)

| composition | Duration | | | | |
| --- | --- | --- | --- | --- | --- |
|  | 8 days | 16 days | 24 days | 32 days | Total |
| 1 | 1136.348 | 1257.197 | 261.143 | 162.62 | 2817.308 |
| 2 | 1101.059 | 1214.481 | 413.208 | 268.57 | 2997.318 |
| 3 | 1048.346 | 1178.497 | 491.381 | 143.07 | 2861.294 |
| 4 | 1013.591 | 783.276 | 319.762 | 230.632 | 2347.261 |
| 5 | 977.294 | 1086.276 | 458.74 | 247.166 | 2769.476 |
| 6 | 907.608 | 1024.207 | 572.514 | 209.99 | 2714.319 |
| 7 | 887.581 | 978.641 | 562.498 | 267.488 | 2696.208 |
| 8 | 1049.367 | 871.492 | 286.738 | 143.767 | 2351.364 |
| 9 | 1015.094 | 1138.764 | 412.164 | 150.227 | 2716.249 |
| 10 | 833.201 | 941.404 | 524.171 | 169.428 | 2468.204 |
| 11 | 774.571 | 917.246 | 577.234 | 28.246 | 2297.297 |
| 12 | 1176.619 | 794.917 | 409.619 | 265.379 | 2646.534 |
| 13 | 1257.694 | 867.215 | 276.918 | 169.54 | 2571.367 |
| 14 | 1178.053 | 976.428 | 291.468 | 172.73 | 2618.679 |
| 15 | 1064.053 | 781.645 | 482.346 | 142.332 | 2470.376 |
| 16 | 1194.981 | 946.376 | 481.367 | 124.251 | 2746.975 |

(d)

| composition | Duration | | | | |
| --- | --- | --- | --- | --- | --- |
|  | 8 days | 16 days | 24 days | 32 days | Total |
| 1 | 1199.637 | 1333.729 | 358.194 | 248.767 | 3140.327 |
| 2 | 1162.181 | 1322.342 | 337.701 | 264.747 | 3086.971 |
| 3 | 1157.537 | 1289.26 | 326.619 | 274.603 | 3048.019 |
| 4 | 876.468 | 994.053 | 379.462 | 208.184 | 2458.167 |
| 5 | 1084.794 | 1198.316 | 469.437 | 76.659 | 2829.206 |
| 6 | 1056.203 | 1156.462 | 342.073 | 226.508 | 2781.246 |
| 7 | 1014.183 | 1109.073 | 447.49 | 135.303 | 2706.049 |
| 8 | 1026.486 | 968.164 | 386.427 | 116.595 | 2497.672 |
| 9 | 1174.816 | 1252.716 | 393.503 | 76.508 | 2897.543 |
| 10 | 1011.049 | 991.27 | 513.573 | 133.316 | 2649.208 |
| 11 | 907.706 | 877.014 | 557.073 | 199.464 | 2541.257 |
| 12 | 1186.516 | 942.816 | 397.29 | 172.139 | 2698.761 |
| 13 | 1176.35 | 991.768 | 376.138 | 250.105 | 2794.361 |
| 14 | 1185.734 | 967.761 | 467.509 | 195.341 | 2816.345 |
| 15 | 1047.54 | 873.438 | 394.368 | 269.015 | 2584.361 |
| 16 | 1264.084 | 971.605 | 412.864 | 197.828 | 2846.381 |

(e)

|  |  |
| --- | --- |
|  |  |
|  |  |

Fig F2. Scatter plot of the testing data set representing observed and model predicted data in T2 type coating

Table S6 Nitrate leaching of different composition of USG with T3 coating (a) age is 1 day (b) age is 2 days (c) age is 3 days (d) age is 4 days (e) age is 5 days

| composition | Duration | | | | |
| --- | --- | --- | --- | --- | --- |
|  | 8 days | 16 days | 24 days | 32 days | Total |
| 1 | 1223.906 | 1319.534 | 255.821 | 131.328 | 2930.589 |
| 2 | 1177.519 | 1287.622 | 244.128 | 93.745 | 2803.014 |
| 3 | 1066.234 | 1184.477 | 342.783 | 104.735 | 2698.229 |
| 4 | 906.929 | 1119.141 | 540.743 | 216.37 | 2783.183 |
| 5 | 1027.492 | 1116.385 | 461.525 | 63.821 | 2669.223 |
| 6 | 1155.199 | 694.199 | 503.862 | 182.543 | 2535.803 |
| 7 | 1297.284 | 747.187 | 380.886 | 114.858 | 2540.215 |
| 8 | 1287.074 | 707.591 | 481.256 | 195.683 | 2671.604 |
| 9 | 950.15 | 1064.124 | 382.463 | 112.845 | 2509.582 |
| 10 | 863.049 | 946.891 | 505.251 | 61.693 | 2376.884 |
| 11 | 974.341 | 877.645 | 299.54 | 191.745 | 2343.271 |
| 12 | 1062.314 | 750.296 | 371.896 | 215.277 | 2399.783 |
| 13 | 976.237 | 922.237 | 318.939 | 150.881 | 2368.294 |
| 14 | 1039.033 | 1030.612 | 174.671 | 114.341 | 2358.657 |
| 15 | 1011.609 | 836.368 | 314.58 | 154.505 | 2317.062 |
| 16 | 837.772 | 894.614 | 452.502 | 53.985 | 2238.873 |

(a)

| composition | Duration | | | | |
| --- | --- | --- | --- | --- | --- |
|  | 8 days | 16 days | 24 days | 32 days | Total |
| 1 | 1259.756 | 1134.57 | 342.7558 | 197.35 | 2934.432 |
| 2 | 1163.964 | 1274.067 | 258.826 | 113.363 | 2810.22 |
| 3 | 1081.871 | 1200.114 | 330.167 | 87.199 | 2699.351 |
| 4 | 925.399 | 1137.611 | 530.96 | 201.667 | 2795.637 |
| 5 | 1128.957 | 1074.331 | 341.433 | 124.345 | 2669.066 |
| 6 | 1175.639 | 714.639 | 496.049 | 169.81 | 2556.137 |
| 7 | 1285.644 | 735.547 | 397.499 | 136.391 | 2555.081 |
| 8 | 1302.604 | 723.121 | 468.533 | 178.04 | 2672.298 |
| 9 | 965.768 | 1079.742 | 369.828 | 95.29 | 2510.628 |
| 10 | 886.981 | 970.823 | 500.93 | 52.452 | 2411.186 |
| 11 | 1202.415 | 831.191 | 517.947 | 188.519 | 2740.072 |
| 12 | 1080.13 | 768.112 | 361.459 | 199.92 | 2409.621 |
| 13 | 963.87 | 909.87 | 334.825 | 171.687 | 2380.252 |
| 14 | 1026.719 | 1018.298 | 190.61 | 135.2 | 2370.827 |
| 15 | 1027.421 | 852.18 | 302.139 | 137.144 | 2318.884 |
| 16 | 854.112 | 910.954 | 440.589 | 37.152 | 2242.807 |

(b)

| composition | Duration | | | | |
| --- | --- | --- | --- | --- | --- |
|  | 8 days | 16 days | 24 days | 32 days | Total |
| 1 | 1364.358 | 1320.193 | 220.411 | 72.75 | 2977.712 |
| 2 | 1174.91 | 1237.985 | 372.532 | 171.345 | 2956.772 |
| 3 | 1180.48 | 1116.86 | 366.522 | 84.191 | 2748.053 |
| 4 | 1247.485 | 1108.928 | 482.849 | 24.917 | 2864.179 |
| 5 | 1015.452 | 1104.345 | 477.738 | 84.954 | 2682.489 |
| 6 | 1280.097 | 1083.333 | 130.704 | 82.335 | 2576.469 |
| 7 | 1351.421 | 1103.978 | 102.194 | 128.34 | 2685.933 |
| 8 | 1366.091 | 1134.632 | 171.549 | 27.351 | 2699.623 |
| 9 | 1005.03 | 1057.196 | 429.708 | 97.443 | 2589.377 |
| 10 | 978.296 | 987.202 | 508.462 | 20.647 | 2494.607 |
| 11 | 1276.167 | 1111.388 | 335.441 | 90.161 | 2813.157 |
| 12 | 1233.712 | 1002.126 | 221.419 | 139.19 | 2596.447 |
| 13 | 1079.197 | 1037.24 | 322.656 | 181.125 | 2620.218 |
| 14 | 1044.753 | 1182.599 | 218.153 | 163.24 | 2608.745 |
| 15 | 1108.497 | 922.113 | 324.007 | 180.976 | 2535.593 |
| 16 | 1089.776 | 1146.937 | 369.631 | 27.178 | 2633.522 |

(c)

| composition | Duration | | | | |
| --- | --- | --- | --- | --- | --- |
|  | 8 days | 16 days | 24 days | 32 days | Total |
| 1 | 1207.452 | 1402.753 | 267.62 | 148.047 | 3025.872 |
| 2 | 1364.929 | 1391.843 | 176.781 | 83.698 | 3017.251 |
| 3 | 1242.976 | 1201.632 | 371.304 | 160.229 | 2976.141 |
| 4 | 1124.716 | 1140.398 | 532.837 | 176.379 | 2974.33 |
| 5 | 1199.88 | 1149.209 | 412.584 | 151.872 | 2913.545 |
| 6 | 1413.783 | 1047.144 | 258.456 | 104.011 | 2823.394 |
| 7 | 1489.684 | 934.191 | 240.166 | 97.081 | 2761.122 |
| 8 | 1424.955 | 1011.336 | 442.599 | 49.776 | 2928.666 |
| 9 | 1131.14 | 1088.086 | 527.304 | 115.642 | 2862.172 |
| 10 | 1058.911 | 1002.1 | 493.449 | 77.258 | 2631.718 |
| 11 | 1416.714 | 859.634 | 370.608 | 190.661 | 2837.617 |
| 12 | 1309.687 | 862.265 | 459.238 | 83.517 | 2714.707 |
| 13 | 1252.586 | 946.893 | 400.705 | 141.383 | 2741.567 |
| 14 | 1270.88 | 1241.378 | 345.173 | 47.724 | 2905.155 |
| 15 | 1289.405 | 936.152 | 356.414 | 55.561 | 2637.532 |
| 16 | 1013.656 | 1080.953 | 549.523 | 94.614 | 2738.746 |

(d)

| composition | Duration | | | | |
| --- | --- | --- | --- | --- | --- |
|  | 8 days | 16 days | 24 days | 32 days | Total |
| 1 | 1288.228 | 1401.241 | 295.285 | 65.729 | 3050.483 |
| 2 | 1322.345 | 1270.753 | 365.752 | 71.976 | 3030.826 |
| 3 | 1269.513 | 1334.951 | 336.7 | 102.142 | 3043.306 |
| 4 | 1107.833 | 1281.644 | 480.639 | 57.324 | 2927.44 |
| 5 | 1181.839 | 1270.731 | 302.814 | 182.473 | 2937.857 |
| 6 | 1277.374 | 1138.322 | 407.649 | 115.352 | 2938.697 |
| 7 | 1262.26 | 1056.599 | 370.548 | 164.386 | 2853.793 |
| 8 | 1357.972 | 1144.414 | 355.2521 | 120.0871 | 2977.725 |
| 9 | 1161.578 | 1221.84 | 420.721 | 132.719 | 2936.858 |
| 10 | 1102.338 | 1161.48 | 453.362 | 132.976 | 2850.156 |
| 11 | 1381.356 | 1181.027 | 292.144 | 19.156 | 2873.683 |
| 12 | 1210.022 | 1030.631 | 321.207 | 292.451 | 2854.311 |
| 13 | 1087.054 | 1183.56 | 499.547 | 147.141 | 2917.302 |
| 14 | 1260.056 | 1367.149 | 265.47 | 187.636 | 3080.311 |
| 15 | 1114.336 | 1034.935 | 453.776 | 165.382 | 2768.429 |
| 16 | 1102.403 | 1150.632 | 508.961 | 122.532 | 2884.528 |

(e)

|  |  |
| --- | --- |
|  |  |
|  |  |

Fig F3. Scatter plot of the testing data set representing observed and model predicted data in T3 type coating
